# Diurnal regulation of cyanogenic glucoside biosynthesis and endogenous turnover in cassava

**DOI:** 10.1002/pld3.38

**Published:** 2018-02-26

**Authors:** Frederik Bøgeskov Schmidt, Seok Keun Cho, Carl Erik Olsen, Seong Wook Yang, Birger Lindberg Møller, Kirsten Jørgensen

**Affiliations:** ^1^ Plant Biochemistry Laboratory Department of Plant and Environmental Sciences University of Copenhagen Copenhagen Denmark; ^2^ VILLUM Research Center “Plant Plasticity” Copenhagen Denmark; ^3^ Center for Synthetic Biology “bioSYNergy” Copenhagen Denmark

**Keywords:** biosynthesis, cytochrome P450, enzyme turnover, linamarin, lotaustralin, *Manihot esculenta*, pathway regulation, transcriptional regulation, UDPG‐dependent glycosyltransferase

## Abstract

Cyanogenic glucosides are present in many plants, including eudicots, monocots, and ferns and function as defence compounds based on their ability to release hydrogen cyanide. In this study, the diurnal rhythm of cyanogenic glucoside content and of transcripts and enzymes involved in their biosynthesis was monitored in cassava plants grown in a glasshouse under natural light conditions. Transcripts of *CYP79D*1, *CYP79D2*,* CYP71E7*/*11,* and *UGT85K5* were at minimal levels around 9 p.m., increased during the night and decreased following onset of early morning light. Transcripts of *UGT85K4* and *HNL10* showed more subtle variations with a maximum reached in the afternoon. Western blots showed that the protein levels of CYP71E7/11 and UGT85K4/5 decreased during the light period to a near absence around 4 p.m. and then recovered during the dark period. Transcript and protein levels of linamarase were stable throughout the 24‐hr cycle. The linamarin content increased during the dark period. In the light period, spikes in the incoming solar radiation were found to result in concomitantly reduced linamarin levels. In silico studies of the promoter regions of the biosynthetic genes revealed a high frequency of light, abiotic stress, and development‐related transcription factor binding motifs. The synthesis and endogenous turnover of linamarin are controlled both at the transcript and protein levels. The observed endogenous turnover of linamarin in the light period may offer a source of reduced nitrogen to balance photosynthetic carbon fixation. The rapid decrease in linamarin content following light spikes suggests an additional function of linamarin as a ROS scavenger.

**Statement of significance**

The biosynthesis and endogenous turnover of cyanogenic glucosides in cassava show strong diurnal regulation at the transcript and protein levels. In addition to their classical function as plant defence compounds, the study points to a role of cyanogenic glucosides as mobilizable storage compounds of reduced nitrogen and carbon and as ROS scavengers.

## 1 INTRODUCTION

Cassava (*Manihot esculenta*) is a highly important stable crop to millions of people in sub‐Saharan Africa. Cassava is mainly grown for its tuberous roots and has many qualities including extreme drought tolerance and a high adaptability to poor soils (El‐Sharkawy, 2004; Nassar, 2001; Nweke, Spencer, & Lynam, 2002). Cassava has gained additional focus as a high‐yielding starch crop suitable for first‐ and second‐generation bioethanol production (Jansson, Westerbergh, Zhang, Hu, & Sun, 2009; Nuwamanya, Chiwona‐Karltun, Kawuki, & Baguma, 2012). Waste from the cassava starch production industry is used as starting material for bioethanol and di‐hydrogen production (Akaracharanya et al., 2011; Cappelletti, Reginatto, Amante, & Antônio, 2011). Cassava contains the potentially toxic cyanogenic glucosides linamarin (2‐β‐D‐glucopyranosyloxy‐2‐methylpropiononitrile) and lotaustralin ([*2R*]*‐* 2‐β‐D‐glucopyranosyloxy‐2‐methylbutyronitrile) (Nartey, 1968). These cyanogenic glucosides are present in all parts of the cassava plant (Nartey, Møller, & Andersen, 1974; Selmar, 1994). Upon tissue disruption caused by a chewing insect or by food processing, the β‐glucosidic linkage in linamarin and lotaustralin is hydrolyzed by the action of linamarase, resulting in the formation of α‐hydroxynitriles, which upon dissociation release toxic hydrogen cyanide and acetone or 2‐butanone either in a spontaneous chemical reaction or catalyzed by an α‐hydroxynitrile lyase (HNL) (Cooke, Blake, & Battershill, 1978; Hughes, Decarvalho, & Hughes, 1994). Based on this hydrolytic bioactivation, cyanogenic glucosides have thus been assigned a major role as plant defence compounds (Gleadow & Moller, 2014; Morant et al., 2008).

**Figure 1:**
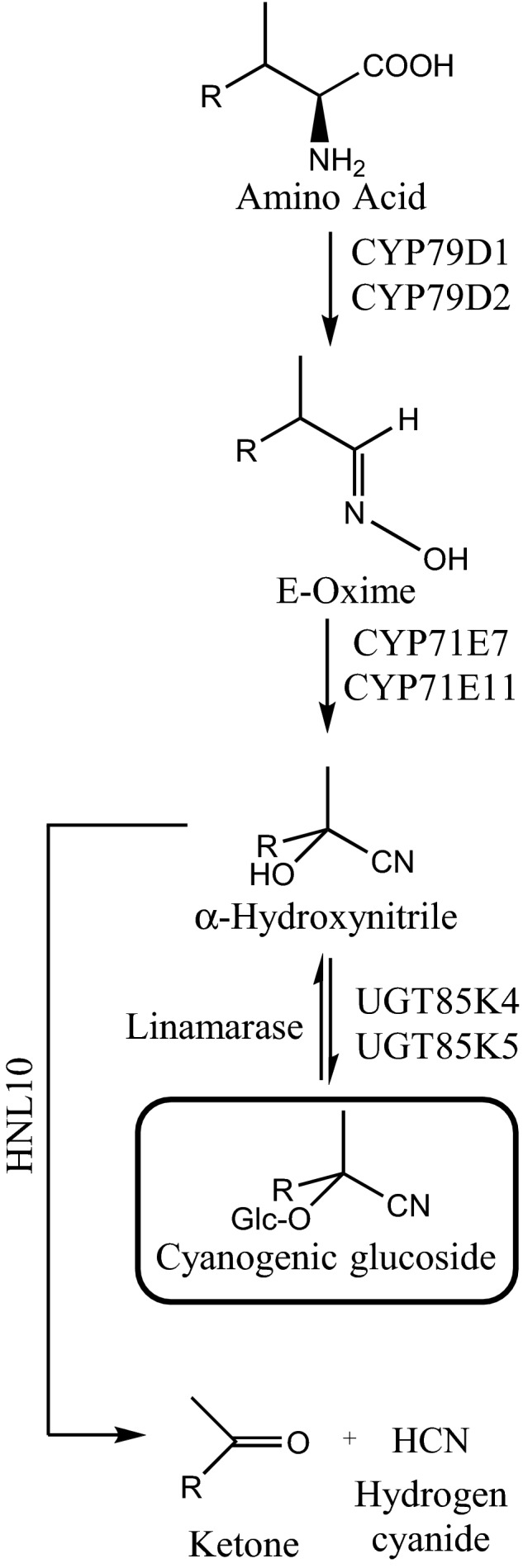
Biosynthesis and hydrolytic bioactivation of the cyanogenic glucosides linamarin (R = methyl) and lotaustralin (R = ethyl) in cassava (*M. esculenta*)

In cassava, the first step of the pathway for biosynthesis of linamarin and lotaustralin (Figure 1) is catalyzed by the cytochrome P450 enzymes CYP79D1 and CYP79D2, which both convert valine and isoleucine to the corresponding oximes (Andersen, Busk, Svendsen, & Møller, 2000). The conversion of the two oximes formed into α‐hydroxynitriles is catalyzed by either CYP71E7 or CYP71E11 (Jørgensen et al., 2010). The hydroxynitriles are then glucosylated by either UGT85K4 or UGT85K5 to produce linamarin and lotaustralin (Kannangara et al., 2011).

Using embryogenic callus cultures, efforts have been undertaken to obtain acyanogenic cassava by RNA interference technology with *CYP79D1* and *CYP79D2* as targets. Transformed plants, in which the cyanogenic glucoside content was reduced to <25% of the cyanide potential of wild‐type plants, had long and slender stems with long internodes and typically did not develop roots. When the nitrogen supply was increased, growth was partly restored (Jørgensen et al., 2005). This suggests that the physiological role of cyanogenic glucosides extends beyond their role as defence compounds (Møller, 2010). The availability of nitrogen and carbon dioxide as well as drought stress affect the cyanogenic glucoside content and growth of cassava (Gleadow, Evans, McCaffery, & Cavagnaro, 2009; Gleadow & Moller, 2014; Rosenthal et al., 2012). The cyanogenic glucoside dhurrin is synthesized and accumulates in the developing sorghum (*Sorghum bicolor*) seed but is turned over in the course of seed maturation so that the mature sorghum seed does not contain dhurrin (Nielsen et al. 2016). When sorghum seeds germinate, dhurrin is rapidly synthesized in the young seedlings reaching levels of 30% of the dry mass in the tip of etiolated seedlings (Busk & Møller, 2002; Halkier & Møller, 1989). The acyanogenic sorghum line *tcd1*, obtained by classical mutagenesis, exhibits slow germination and reduced height of adult plants compared to wild‐type plants (Blomstedt et al., 2012). In the rubber tree (*Hevea brasiliensis*), the cyanogenic glucoside content is influenced by time of day, light and by latex tapping (Kongsawadworakul et al., 2009).

Recently, endogenous turnover of cyanogenic glucosides was demonstrated in cassava, sorghum, and almonds (Nielsen et al., 2016; Picmanova et al., 2015). This endogenous turnover pathway operates with amides, carboxylic acids, and anitriles as intermediates. Instead of releasing toxic hydrogen cyanide, the endogenous turnover pathway results in release of ammonia. This supports a general function of cyanogenic glucosides as storage forms of reduced nitrogen and carbon (Nielsen et al., 2016; Picmanova et al., 2015).

In this study, we demonstrate that the production and accumulation of cyanogenic glucosides follow a strong diurnal rhythm with turnover of a significant fraction of the cyanogenic glucosides in the light and resynthesis during the night. The synthesis of linamarin is controlled both at the transcript and protein levels.

## 2 RESULTS

To identify factors possibly involved in regulating biosynthesis and endogenous turnover of cyanogenic glucosides at the molecular level, an initial in silico analysis of the promoter regions of the genes encoding enzymes involved in cyanogenic glucoside biosynthesis and hydrolytic bioactivation was carried out. Based on the findings from the in silico study, an experiment investigating the diurnal variation of cyanogenic glucoside metabolism was set up. Throughout a 25‐hr period, cassava plants grown in a glasshouse and subjected to natural light conditions were sampled every single hour. Plants were analyzed for their cyanogenic glucoside content and the presence of transcripts and proteins known to be involved in the biosynthesis and hydrolytic bioactivation of cyanogenic glucosides.

### 2.1 In silico analysis of gene promoter regions

**Figure 2:**
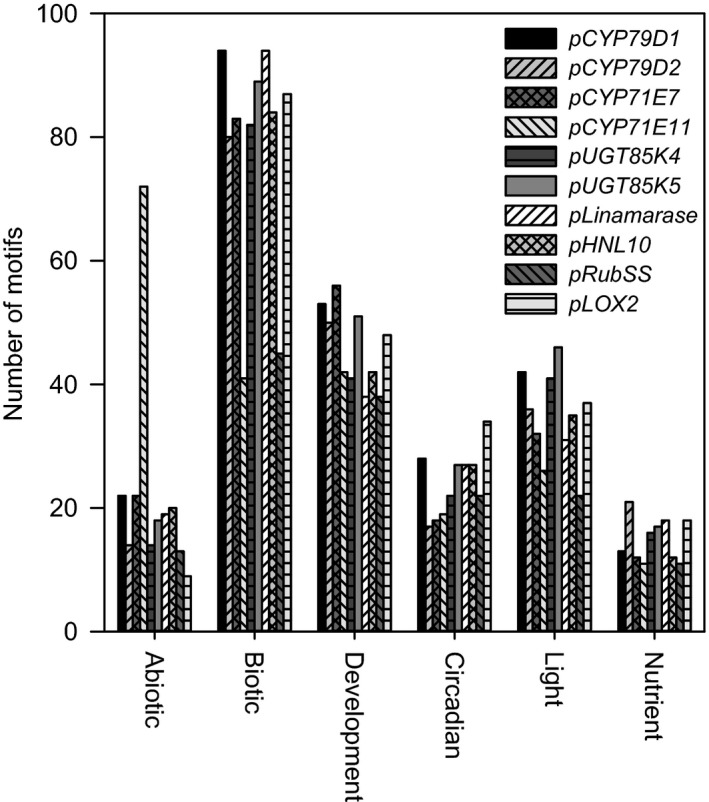
Number of transcription binding factor motifs related to abiotic and biotic stresses, circadian rhythm, light responses, development as well as nutrient stress present in the promoter region of genes encoding enzymes involved in the biosynthesis and hydrolytic bioactivation of linamarin and lotaustralin in cassava (*M. esculenta*)

In silico analysis of the promoter regions of the genes encoding the enzymes involved in biosynthesis and hydrolytic bioactivation of cyanogenic glucosides was carried out to gain preliminary insight into possible key factors involved in their regulation. Analysis was restricted to the presence of transcription factor binding sites (TFBSs) within a promoter sequence of 1,500 bp encompassing the typical core promoter elements involved in basal transcription such as Transcription Start Sites (TSS), multiple TATA boxes and CAAT boxes. The analysis revealed a wide range of regulatory motifs putatively related to abiotic and biotic (including jasmonic acid) stresses, development (including development related hormones), circadian rhythm, light, and nutritional status (Figure 2, Table S1). In the abiotic stress category, motifs related to ABA and dehydration‐related stress factors were predominant. In this category, *pCYP71E11* stood out with more than three times the number of motifs compared to any of the other promoters analyzed. The promoters of the genes encoding the two hydrolytic enzymes contained a number of abiotic resistance‐related motifs comparable to those found in the genes encoding the biosynthetic enzymes with the exception of *pCYP71E11*. Biotic stress‐related motifs included all motifs related to jasmonate, defence, or pathogen response. In this category, *pCYP71E11* stood out as containing only half the number of motifs compared to *pCYP71E7* and other promoters of the genes encoding enzymes in the metabolism of cyanogenic glucosides. Regulatory motifs associated with development included sequence motifs responding to development‐related hormones and tissue‐specific expression. The promoters of the genes encoding biosynthetic enzymes had a higher number of motifs for at least one of the homologs compared to the genes encoding hydrolytic enzymes. Motifs related to circadian regulation were found in all promoters with most found in *pCYP79D1*,* pUGT85K5,* and the promoters of the hydrolytic genes. *pCYP79D2* showed very few motifs compared to *pCYP79D1*, thus indicating different roles for the two homologs with regard to circadian regulation. In the light‐related motifs category, *pCYP79D1* and the two *pUGTs* contained the highest number of motifs. This combined with the circadian category indicated that the regulation of *pCYP79D1* and the *pUGTs* is influenced by light. *pCYP79D2*, the two *pUGTs,* and *pHNL10* had similar or higher number of motifs related to nutrient‐related regulation than the rest of the analyzed promoters. *pCYP79D1* contained in general more motifs than its *pCYP79D2* homolog. Whereas *pCYP71E11* exhibited a uniquely high number of abiotic stress‐related motifs, *pCYP71E7* contained a high number of biotic stress‐related motifs. For comparisons, the promoters of *Rubisco small subunit* (*pRubSS*) and *LOX2* (*pLOX2*) were analyzed as representative of genes encoding enzymes related to photosynthesis and biotic stress, respectively (Jung et al., 2007; Spoel et al., 2003). *pRubSS* contained a small number of motifs and was in the lowest half in all categories in agreement with consistent expression and poor stress sensitivity of *Rubisco small subunit. pLOX2* contained a number of motifs comparable to the categories and general trend found in the promoters for the genes encoding enzymes in cyanogenic glucoside metabolism, except that *pLOX2* had a higher number of motifs in the circadian category and fewer motifs in the abiotic stress category.

### 2.2 Cyanogenic glucoside content

The stoichiometric ratio between the two cyanogenic glucosides linamarin and lotaustralin present in cassava is general around 90:10 (Lykkesfeldt & Møller, 1994; Nartey, 1968). In this study, only data for linamarin are presented, as the calculated ratio was consistent and close to the general ratio. An ANOVA test was performed for the data set and showed *p* < .001, *F* > *F*_crit_.

**Figure 3:**
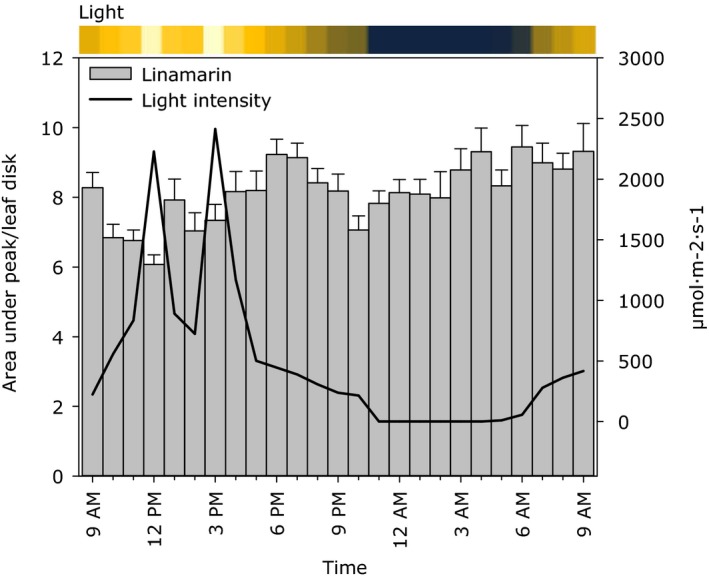
Diurnal variation of linamarin content in the second fully unfolded leaf of 3‐month‐old cassava (*M. esculenta*) plants measured by LC‐MS and displayed as area under peak per 10 mm leaf disk calibrated against internal standards. The measured light intensities are shown as a line graph and converted to a color scale presented above the plot. Bars indicate *SE*,* n* = 10. ANOVA test: *p* < .001, *F* > *F*_crit_

LC‐MS analysis demonstrated that the linamarin content varied during the day and night cycle with a difference of 35% between the highest and the lowest levels (Figure 3). From 9 a.m. until noon, the linamarin content was found to decrease moderately. The content increased in the afternoon until 7 p.m., where it again decreased reaching a minimum at 10 p.m. The linamarin content increased slowly through the night and early morning (Figure 3). This demonstrated a diurnal variation in linamarin content. The plants were grown in a glasshouse and recorded spikes in incoming solar irradiation before and after 1 p.m. co‐occurred with a reduction in linamarin content. Similar observations were made in other experiments (Figures S1 and S2). This indicates that linamarin content is regulated by a diurnal rhythm as well as negatively influenced by light strikes.

### 2.3 Protein levels of enzymes catalyzing cyanogenic glucoside biosynthesis and hydrolytic bioactivation

**Figure 4:**
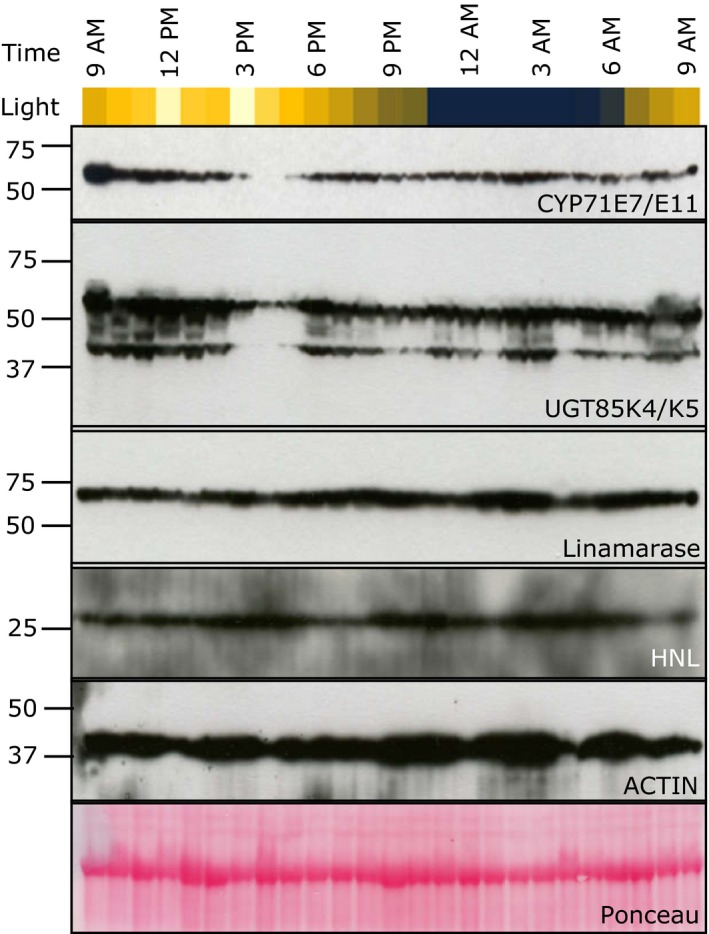
Diurnal variation of protein content of CYP71E7/11, UGT85K4/5, linamarase, and α‐hydroxynitrile lyase (HNL) as monitored by Western blot using specific antibodies. β‐Actin was used as reference protein and monitored using a specific antibody and by Ponceau stain. Color scale below the hour scale indicates light intensities

The protein levels of CYP71E7/11 and UGT85K4/5 displayed oscillatory dynamics throughout the entire light period as determined by immunoblot analysis (Figure 4). The antibodies used do not distinguish between the two homologs of each protein. The levels of the CYP71E7/11 and UGT85K4/5 proteins were at their highest around 9 a.m. and decreased to almost nondetectable levels between 3 p.m. and 5 p.m. This synchronization of protein expression pattern and/or degradation indicated that the proteins are coregulated by a yet unknown factor or factors. Several immune responsive protein bands were observed using the UGT85 antibody. When the antibody was tested against crude protein extracts of the first fully unfolded leaf of cassava plants grown under at lower light intensities, a single protein band was observed (Kannangara et al. 2011). We therefore conclude that the additional bands observed in this study represent proteolytic degradation products of the UGT85K4/5 proteins. In contrast to the CYP71E7/11 and UGT85K4/5 proteins, linamarase and HNL did not show any distinct variation in their levels as compared to the β‐actin baseline. The reduction in CYP71E7/11 and UGT85K4/5 protein levels at the start of the light period occurs simultaneously with the decrease in cyanogenic glucoside content. However, the protein levels drop to a minimum approximately 3 hr later than the decrease in linamarin content. The linamarin concentration is thus controlled by other factors in addition to de novo protein synthesis, for example, post‐translational modifications. The level of the linamarin‐degrading enzymes linamarase and HNL remained constant throughout the tested period but it cannot be excluded that their catalytic activities varied, for example, based on post‐translational modifications. More likely though is the operation of endogenous turnover pathways for which the intermediates are known but none of the enzymes and genes involved have been identified (Nielsen et al., 2016, Picmanova et al. 2015).

### 2.4 Transcript levels of genes encoding enzymes catalyzing cyanogenic glucoside biosynthesis and hydrolytic bioactivation

Transcript levels of the genes involved in the biosynthesis and hydrolytic bioactivation of cyanogenic glucosides (*CYP79D1*,* CYP79D2*,* CYP71E7/E11*,* UGT85K4*,* UGT85K5*,* linamarase,* and *HNL10*) were quantified by real‐time PCR and normalized against the reference genes *GAPDH* and β*‐actin*. As a positive control for diurnal variation, the gene encoding the Rubisco small subunit (Phytozome: Manes.05G137400.1) was included in the transcript analysis. In general, the expression of the majority of the analyzed genes showed a distinct diurnal variation and rapid changes in transcript levels within the hour.

**Figure 5:**
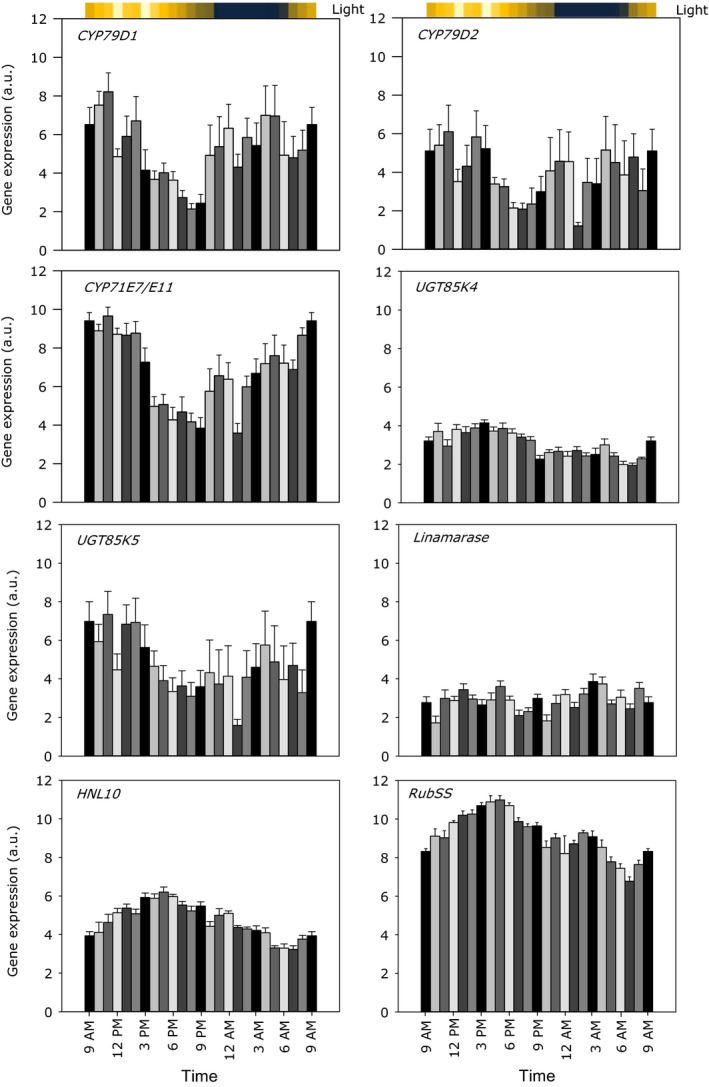
Diurnal variation of the levels of the gene transcripts encoding the enzymes in the biosynthesis and hydrolytic bioactivation of linamarin and lotaustralin in cassava (*M. esculenta*) measured by real‐time qPCR with β‐Actin and GAPDH as reference genes. Rubisco small subunit (RubSS) is included as control of reaction to light. Color scale above the plots indicates the differences in light intensities measured at the different time points. Bars indicate *SE*,* n* = 10

The transcript levels of the cytochrome P450 encoding genes involved in the biosynthesis of cyanogenic glucosides in cassava exhibited similar overall transcription patterns (Figure 5). In the light period from noon until evening, the transcript levels of *CYP79D1*,* CYP79D2,* and *CYP71E7/11* decreased by more than 50%. An equivalent rise then occurred during the following dark period. *CYP79D2* showed lower expression level in comparison with *CYP79D1*. A clear drop in *CYP79D1* and *CYP79D2* transcript levels was observed at peak light intensity at noon. A similar drop was observed around midnight which could not be related to any specific event. The transcript levels of *CYP71E7/11* were very similar to the transcript profile of *CYP79D1*, but differences in expression between the *CYP71E7* and *CYP71E11* cannot be excluded because they were not monitored separately.

Contrary to the two *CYP79D* homologs, the *UGT85K4* and *UGT85K5* homologs did not show the same diurnal expression profile (Figure 5). The expression profile of *UGT85K5* exhibited a similar expression profile as *CYP79D1/2*, whereas *UGT85K4* was more stably expressed with a maximum of gene expression at the time period where *UGT85K5* showed the lowest expression. *UGT85K4* showed higher expression in the light compared to dark. Except for a single time point, the expression level of *UGT85K4* was always lower than that of *UGT85K5* (Figure 5).

Linamarase catalyzes the first step in the hydrolytic bioactivation of cyanogenic glucosides and did not show any obvious diurnal changes in its transcript pattern. *HNL10* exhibited a more distinct expression pattern characterized by an increase during the light period and a decrease again from the evening until the light period started. This co‐occurs with the decrease in cyanogenic glucoside content, indicating that HNL10 might be involved in the hydrolysis of cyanogenic glucosides during the light period besides its direct role in hydrogen cyanide release.

## 3 DISCUSSION

In this study, the effect of the diurnal rhythm on the metabolism and content of the cyanogenic glucoside linamarin in cassava was investigated using plants grown in a glasshouse.

The content of cyanogenic glucosides has previously been reported to vary between vegetative cassava clones propagated from stem cuttings, even when grown under identical and sterile conditions in a climate chamber (Jørgensen et al., 2005). As for other bioactive natural products, the accumulation of cyanogenic glucosides is controlled by their rate of biosynthesis relative to the rate of hydrolytic bioactivation and endogenous turnover. Deciphering how these processes are controlled by variations of transcript and protein levels and by post‐translational modifications is highly complex. Unfortunately, the enzymes and genes involved in the endogenous turnover pathway of cyanogenic glucosides are yet to be identified. The endogenous turnover products of linamarin are linamarin amide, linamarin acid, and linamarin anitrile, and these may all be detected in cassava leaves but in levels representing around 0.1% of that of linamarin (Nielsen et al., 2016; Picmanova et al., 2015). The low amounts of endogenous turnover products most likely reflect their rapid further metabolism and incorporation into primary metabolism.

The results demonstrated that the levels of the biosynthetic enzymes CYP71E7/11 and UGT85K4/5 decreased upon the onset of morning light with minimum levels between 4 p.m. and 6 p.m. A similar decrease was observed for the transcripts of *CYP79D1, CYP79D2, CYP71E7/11,* and *UGT85K5*. The protein and transcript levels for these increased during the dark period. A similar recovery of the linamarin levels was observed in the dark period, whereas the pattern in the light was rather complex. Despite rapid changes in protein and transcript levels, only moderate changes were observed on metabolite level. The changes in linamarin levels related to the diurnal rhythms were overlaid by decreases caused by abrupt increases in light intensity caused by changes from a cloudy to a clear sky (Figure 3). Prior to this experiment, the experiment was repeated twice in minor scale, and the same trends were observed in all three experiments (Figure 3, Figures S1 and S2). Similar results have been reported in green leaves of *Olinia ventosa*, where exposure to strong light resulted in the conversion of prunasin into its corresponding amide (Sendker & Nahrstedt, 2009). The amide formation observed under high light irradiation may not necessarily reflect the operation of the endogenous turnover pathway. It is most likely assigned to the ability of cyanogenic glucosides to sequester reactive oxygen species (ROS) in a nonenzymatic Radziszweski process by which the nitrile functional group is converted into an amide group (Møller, 2010; Sendker & Nahrstedt, 2009). In rubber tree (*Hevea braziliensis*), more than twofold decrease in linamarin content was observed during the light period, and the linamarin content of shade leaves was higher than in leaves exposed to direct light (Kongsawadworakul et al., 2009). In developing sorghum seedlings, the rise in the content of the cyanogenic glucoside dhurrin was much higher during the night compared to the light period (Adewusi, 1990). The observed accumulation of proteolytic degradation products of the UGT85K4/5 proteins may be related to the degradation of CYP79D1/D2 and CYP71E7/11 proteins during the light period. A surplus of intact functionally active UGT85K4/5 proteins not bound within a metabolon may be highly damaging to the cells because these UGT′s when tested as free soluble enzymes in in vitro assays have a broad substrate specificity and thus may catalyze a number of undesired glucosylation reactions (Jørgensen et al. 2005; Kannangara et al., 2011). Detailed studies in the rubber tree further suggested that linamarin present in the inner bark of the rubber tree was degraded between latex tapping because linamarin served as an endogenous source of reduced nitrogen and carbon used to build‐up and maintain the rubber‐producing machinery (Kongsawadworakul et al., 2009). The rapid decreases in linamarin content in the light period may thus reflect a function of linamarin as a ROS quencher as well as a reservoir of reduced nitrogen and carbon available to balance the high rate of carbon dioxide fixation and to meet demands in primary metabolism. Cassava plants grown in soil with reduced nitrogen content have been shown to have a reduced cyanogenic glucoside content compared to plants grown in nitrogen replete soil (Gleadow et al., 2009). In this study, such a function may also explain the decrease in linamarin content from 6 p.m. to 9 p.m., a time period where the levels of the biosynthetic enzymes and their transcripts increase, but where carbon skeletons and reduced nitrogen might be limiting. In cassava, the function of linamarin and lotaustralin as storage compounds of reduced nitrogen and carbon and as quenchers of ROS and diurnal changes in their rate of biosynthesis may explain the observed changes in linamarin content during the light period (Figure 3).

The levels of the hydrolytic enzymes remained constant throughout the diurnal cycle as monitored by specific antibodies. At the transcript level, linamarin transcription remained constant, whereas in case of HNL, it increased in the light period followed by a decrease during the night period. The capacity for hydrolytic bioactivation of linamarin with concomitant release of toxic hydrogen cyanide to gain insect resistance was thus maintained throughout the entire diurnal cycle in spite of the downregulation of linamarin biosynthesis and reduced linamarin content during the light period where most cassava folivores are active (FAO, 2013). This implies that the amount of linamarin synthesized during the night period is high enough to fulfill its purposes in insect defence, as a ROS scavenger and supplier of reduced nitrogen and carbon to primary metabolism. The transcript levels of the genes encoding linamarin biosynthetic enzymes were shown to undergo rapid changes. This demonstrates that fine‐tuning of the expression of these genes is required to enable the plant to respond rapidly to environmental stress factors. Cytochrome P450s catalyze key steps in the biosynthesis of linamarin (Andersen et al., 2000). Their transcripts show a highly similar regulatory pattern. The same transcript pattern is observed for *UGT85K5*, whereas the transcript profile for *UGT85K4* remains nearly constant throughout the diurnal cycle. The differences in transcript regulation could indicate that the two UGT85K homologs have different roles and are responsible for reacting to different stress factors as also indicated by the promoter analysis. Except for *CYP79D1*, the genes involved in the biosynthesis of linamarin and lotaustralin in cassava have been found to cluster within a 100 kb region of the cassava genome (Takos et al., 2011). This may facilitate coregulation of their expression as observed for all analyzed genes except *UGT85K4*. Exposure of the cassava plants to herbivores and pests is envisioned to confer yet another layer of modulation of the expression of the genes encoding the enzymes catalyzing biosynthesis and hydrolytic bioactivation to optimize the *in planta* function of cyanogenic glucosides to combat insect attacks by release of toxic hydrogen cyanide (Gleadow and Møller, 2014) (Figure S2). It has been hypothesized that the high levels of reactive oxygen species produced as a response to fungal attack may denature the oxygen‐sensitive CYP71E enzyme resulting in the formation of an (E)‐oxime (Møller 2010). (E)‐Oximes are antifungicides. It is possible that the gene pairs encoding the biosynthetic enzymes in cassava are differentially expressed or differentially induced by abiotic or biotic stresses as also suggested by the observed uneven distribution of transcription factor binding cis‐elements in their promoters. These differences are pronounced in relation to all stress factors implying that the biosynthesis of linamarin and lotaustralin could be composed of a constitutive pathway supplemented by increased biosynthesis in response to stress factor and thus giving the plant a high level of plasticity (Figure 2).

The classical function of cyanogenic glucosides is their role as phytoanticipins in a two‐component insect defence system which by the action of β‐glucosidases upon cell disruption results in the detonation of a hydrogen cyanide bomb (Conn, 1969; Gleadow & Moller, 2014; Morant et al., 2008). This study suggests that their role beside the defence is much more diversified, including possible functions as ROS scavengers and as storage compounds for reduced nitrogen and carbon.

## 4 EXPERIMENTAL PROCEDURES

### 4.1 Plant material

Cassava (*M. esculenta*) plants of the cultivar TME12 were obtained from stem cuttings provided by the International Institute of Tropical Agriculture (IITA), Ibadan, Nigeria. The 3‐month‐old cassava plants (50–70 cm tall) used in all experiments were clonally propagated using 5‐ to 8‐cm‐long stem cuttings from older plants excised more than 10 cm below the apex and carrying at least two nodes. The cuttings were sprouted in 12‐cm‐diameter pots with nutrient replete soil and watered regularly. The plants were grown in a glasshouse with 16 hr light/28°C and 8 hr dark/25°C cycle. During the light cycle, the natural light was augmented with artificial light (200 μmol·m^−2^·s^−1^).

### 4.2 In silico promoter analysis

The 1,500 bp regions upstream of the ATG start codon of *CYP79D1*,* CYP79D2*,* CYP71E7*,* CYP71E11*,* UGT85K4*,* UGT85K5*,* linamarase*,* HNL10*,* Rubisco small subunit,* and *LOX2* were sourced from the Cassava v4.1 genome sequence (Prochnik et al., 2012) using blast search. In silico analysis for the presence of regulatory elements in the promoters was carried out using Matinspector (Genomatix GmbH).

### 4.3 Diurnal variation

Diurnal variation in cyanogenic glucoside metabolism was monitored at the metabolite, transcript, and protein level throughout a 25‐hr period. Each hour, the second fully unfolded leaf was harvested from each of ten plants adding up to 250 plants harvested in total. At the same time point, the light intensity was measured. Leaf disks sampled for metabolite profiling were analyzed immediately after harvest, whereas leaf samples for transcript and protein analysis were frozen in liquid nitrogen and kept at −80°C until analyzed.

### 4.4 Metabolite analysis

A single leaf disk (⌀ = 10 mm) was excised from the center part of each of ten leaves, excluding the midrib. To avoid loss of labile metabolites, each single leaf disk (10 biological replicates) was extracted in boiling 85% MeOH (300 μl, 3 min, 1.5 ml sealed screw‐cap tube) immediately after harvest. After boiling, the tubes were chilled on ice and the liquid fraction transferred to a glass vial and stored at 5°C and analyzed within 4 days. Aliquots (15 μl) were diluted with 135 μl water and filtered (0.45 μm 96‐well filter plate, 2,000 *g*, 10 min) and hereafter transferred to a new glass vial for LC‐MS and analyzed as in Jørgensen et al. (2005). The data were analyzed using DataAnalysis 4.0 (Bruker Daltonics GmbH), and statistical analysis was performed in Excel 2010 (Microsoft).

### 4.5 Transcript analysis

The transcript levels of genes involved in the biosynthesis and degradation of cyanogenic glucosides and of reference genes were determined by real‐time PCR. Two leaf disks (⌀ = 10 mm, 10 biological replicates) were excised from the center part of each of ten leaves, excluding the midrib. Total RNA was extracted with the Sigma‐Aldrich Spectrum™ Plant Total Kit according to manufacturer's instructions, except that the column was washed with 300 μl Wash Buffer I after the binding step, and the bound RNA was DNase treated with QIAGEN RNase‐free DNase according to manufacturer's instructions. The concentration of the isolated RNA was measured with a NanoDrop^®^ ND‐1000 Spectrophotometer and the RNA stored at −80°C. cDNA was synthesized from the RNA (900 ng) using the Bio‐Rad iScript™ cDNA synthesis kit according to manufacturer's description. Real‐time PCR was performed by TATAA Biocenter (Göteborg, Sweden) with assays targeting *CYP79D1* (fwd: 5′‐atcactctcaaggattcagacgg‐3′, rev: 5′‐ctcttgcacaggctttgacatagt‐3′), *CYP79D2* (fwd: 5′‐tggcctgatctcaagtacgac‐3′, rev: 5′‐gagtaagctcatcgatattctcac‐3′), *CYP71E7* (fwd: 5′‐tgttgatgtcttgattgggttga‐3′, rev: 5′‐gtatttgatcttctccacctcttttc‐3′), *UGT85K4* (fwd: 5′‐aggcttgttcctgcgtgag‐3′, rev: 5′‐tggagcactcacagttcaca‐3′), *UGT85K5* (fwd: 5′‐gccatccttcaacacaagacta‐3′, rev: 5′‐ggcaataaccatctccaatacag‐3′), *linamarase* (fwd: 5′‐aaactgatagtggtgttaatgcgact‐3′, rev: 5′‐gaagtgcctctccatttggttg‐3′), *HNL10* (fwd: 5′‐gacaccgttcatagcccatcttac‐3′, rev: 5′‐cagtgatcccttcctcattaccat‐3′), *Rubisco small subunit* (fwd: 5′‐gccctgctcaggctactatg‐3′, rev: 5′‐gctcccttgtaagtggtgga‐3′), β*‐Actin* (fwd: 5′‐atgttgcccttgactatgaacag‐3′, rev: 5′‐aaggtccttcctgatatccacat‐3′), and *GAPDH* (fwd: 5′‐cagcagcgaccattatcttgtc‐3′, rev: 5′‐tccactcggcatctgcgtac‐3′). Data were analyzed using GenEx Pro (MultiD) and exported for visualizing in SigmaPlot (Scientific Computing).

### 4.6 Protein analysis

Frozen leaf disks (two pieces) were homogenized (2 min, 25 Hz, liquid nitrogen) using a mixer mill (Retsch MM 301, Germany). Each extract was mixed with SDS sample buffer (200 μl, 10 min, boiling) and applied to a 12% Criterion™ XT Bis‐Tris Gel (Bio‐Rad, USA). After electrophoretic separation, the proteins were transferred to PVDF membranes, 100V 30 min in transfer buffer (25 mM Tris, 192 mM glycine, 0.1% (w/v) SDS, pH 8.3) (Bio‐Rad). Unspecific binding was prevented by treatment of the membranes with blocking solution (5% skim milk in 1× PBS with 0.1% Tween‐20, 1 hr, RT) and followed by incubation (ON, 4°C) with anti‐CYP71E (1:2,000), anti‐UGT85 (1:2,000), anti‐linamarase (1:2,000), anti‐HNL (1:2,000), or anti‐actin (1:3,000; Agrisera, Umeå, Sweden). Membranes were washed (15 min) with 1× PBST and incubated (1 hr, RT) with HRP‐conjugated secondary antibody (Dako, Denmark). The immunoblots were visualized with SuperSignal West Dura Extended Duration Substrate (Thermo, USA).
