# Supplementary material for: Diurnal regulation of cyanogenic glucoside biosynthesis and endogenous turnover in cassava
Source: Plant Direct. 2018 Feb 26;2(2):e00038. doi: 10.1002/pld3.38 (PMC6508492; doi:10.1002/pld3.38)
Supplement: Supplementary file 1 [file PLD3-2-e00038-s001.docx]

Table S1: Table of TFBS motifs found in the promoter regions of *CYP79D1*, *CYP79D2*, *CYP71E7*, *CYP71E11*, *UGT85K4*, *UGT85K5*, *Linamarase*, *HNL10*, *RubSS* and *LOX2*. Promotor regions were defined as 1500kb upstream of ATG sites of the genes and were analysed with Genomatix MatInspector. Motif matrix families were categorised according to the literature in six major categories related to abiotic, biotic, development, circadian, light and nutrient responsive elements.

| **Motif** | **pCYP79D1** | **pCYP79D2** | **pCYP71E7** | **pCYP7E11** | **pUGT85K4** | **pUGT85K5** | **pLinamarase** | **pHNL10** | **pRubSS** | **pLOX2** | **Description** | **Category** |
| --- | --- | --- | --- | --- | --- | --- | --- | --- | --- | --- | --- | --- |
| ABRE | 2 | 2 | 4 | 0 | 0 | 1 | 0 | 2 | 2 | 0 | Abiotic, ABA, Drought, Salt stress | Abiotic |
| CNAC | 0 | 2 | 2 | 0 | 1 | 1 | 0 | 0 | 1 | 1 | Abiotic stress, Calcium |  |
| DPBF | 0 | 0 | 0 | 1 | 0 | 0 | 0 | 0 | 0 | 0 | Drought, Cold, ABA, GA |  |
| DREB | 1 | 0 | 0 | 1 | 0 | 0 | 2 | 1 | 0 | 1 | Dehydration, Cold |  |
| NACF | 4 | 3 | 9 | 1 | 7 | 7 | 2 | 3 | 1 | 3 | Abiotic, ABA |  |
| NTMF | 6 | 3 | 4 | 7 | 2 | 1 | 4 | 6 | 4 | 2 | Abiotic, GA |  |
| PNRE | 0 | 0 | 0 | 2 | 2 | 0 | 2 | 0 | 4 | 1 | Abiotic, Nitrate, Drought |  |
| PSPE | 4 | 1 | 0 | 55 | 0 | 0 | 1 | 3 | 0 | 0 | Abiotic, Heat, SA |  |
| SALT | 0 | 0 | 0 | 0 | 0 | 0 | 1 | 0 | 1 | 0 | Abiotic, Salt, Drought |  |
| SWNS | 5 | 3 | 3 | 5 | 2 | 8 | 7 | 5 | 0 | 1 | Abiotic, Drought, ABA, Auxin, BR |  |
| Sum | 22 | 14 | 22 | 72 | 14 | 18 | 19 | 20 | 13 | 9 |  |  |
| AHBP | 41 | 41 | 31 | 16 | 42 | 43 | 45 | 39 | 20 | 48 | Defense, SA, ABA | Biotic |
| AHLF | 16 | 6 | 10 | 3 | 6 | 11 | 22 | 15 | 4 | 14 | Defense, Immune response |  |
| ASRC | 3 | 1 | 0 | 2 | 2 | 3 | 0 | 0 | 5 | 0 | Defense, Immune response |  |
| BRRE | 0 | 1 | 3 | 0 | 0 | 0 | 0 | 1 | 0 | 0 | Defense, BR |  |
| CE1F | 0 | 0 | 1 | 0 | 0 | 1 | 1 | 0 | 0 | 0 | Defense, ABA, JA, Ethylene |  |
| CGCG | 0 | 1 | 0 | 0 | 3 | 1 | 0 | 0 | 0 | 0 | Defense |  |
| DOFF | 6 | 9 | 8 | 3 | 11 | 9 | 7 | 8 | 3 | 4 | Defense, Auxin, JA, SA |  |
| EINL | 2 | 0 | 1 | 0 | 1 | 1 | 0 | 3 | 0 | 1 | Defense, Ethylene, Iron ion |  |
| FORC | 0 | 0 | 1 | 0 | 0 | 0 | 0 | 0 | 0 | 0 | Defense |  |
| GBOX | 6 | 3 | 5 | 2 | 0 | 2 | 5 | 2 | 1 | 1 | Defense, SA, GA |  |
| GCCF | 0 | 1 | 0 | 0 | 0 | 0 | 0 | 1 | 0 | 0 | Defense, JA, SA, Auxin, ABA, light |  |
| JARE | 0 | 0 | 1 | 0 | 0 | 1 | 0 | 1 | 0 | 4 | Defense, JA |  |
| MIIG | 8 | 5 | 6 | 2 | 4 | 0 | 1 | 3 | 3 | 4 | Defense, ABA, Auxin, Ethylene, GA, JA, SA |  |
| MYBL | 4 | 9 | 10 | 5 | 9 | 10 | 8 | 5 | 4 | 9 | Defense, Auxin, JA, GA |  |
| OCSE | 4 | 1 | 4 | 5 | 2 | 3 | 4 | 2 | 2 | 2 | Defense, SA |  |
| WBXF | 2 | 1 | 1 | 2 | 2 | 2 | 1 | 2 | 1 | 0 | Defense, ABA, SA, JA |  |
| WTBX | 2 | 1 | 1 | 1 | 0 | 2 | 0 | 2 | 2 | 0 | Defense, JA, SA |  |
| Sum | 94 | 80 | 83 | 41 | 82 | 89 | 94 | 84 | 45 | 87 |  |  |
| AP2L | 1 | 2 | 1 | 0 | 0 | 0 | 0 | 0 | 0 | 0 | Development | Development |
| AREF | 0 | 0 | 1 | 0 | 0 | 2 | 0 | 0 | 0 | 0 | Development, ABA, Auxin |  |
| ARF3 | 0 | 0 | 1 | 0 | 0 | 0 | 2 | 1 | 0 | 0 | Development, Auxin |  |
| CAAT | 4 | 0 | 0 | 2 | 0 | 0 | 0 | 2 | 2 | 2 | Development, ABA |  |
| E2FF | 0 | 0 | 1 | 0 | 0 | 0 | 0 | 0 | 0 | 1 | Development, Auxin |  |
| EREF | 0 | 0 | 0 | 0 | 0 | 0 | 1 | 1 | 0 | 1 | Development, Ethylene, Carbohydrate |  |
| ERSE | 0 | 0 | 0 | 0 | 0 | 0 | 1 | 0 | 0 | 0 | Development |  |
| FLO2 | 1 | 0 | 0 | 1 | 0 | 0 | 2 | 2 | 1 | 2 | Development |  |
| GAGA | 2 | 0 | 0 | 0 | 0 | 0 | 0 | 1 | 0 | 0 | Development, Ethylene |  |
| GARP | 1 | 1 | 0 | 2 | 1 | 1 | 0 | 0 | 2 | 3 | Development, Ethylene |  |
| KAN1 | 4 | 7 | 5 | 5 | 5 | 7 | 6 | 3 | 5 | 1 | Development |  |
| L1BX | 18 | 13 | 25 | 5 | 12 | 21 | 11 | 10 | 4 | 18 | Development |  |
| LBDF | 0 | 1 | 0 | 0 | 0 | 0 | 0 | 0 | 0 | 0 | Development |  |
| LEGB | 1 | 3 | 1 | 1 | 2 | 0 | 0 | 2 | 0 | 1 | Development, ABA, Auxin |  |
| LFYB | 1 | 0 | 0 | 0 | 0 | 0 | 0 | 1 | 1 | 0 | Development, GA |  |
| LICM | 0 | 0 | 0 | 0 | 1 | 0 | 0 | 0 | 0 | 0 | Development |  |
| MADS | 6 | 12 | 9 | 13 | 5 | 8 | 9 | 9 | 8 | 7 | Development, Auxin, GA |  |
| MSAE | 0 | 0 | 2 | 1 | 1 | 0 | 0 | 1 | 2 | 0 | Development |  |
| NCS1 | 1 | 1 | 3 | 5 | 2 | 5 | 3 | 4 | 5 | 2 | Development, Nodule |  |
| NCS2 | 0 | 0 | 0 | 1 | 1 | 0 | 0 | 0 | 0 | 0 | Development, Nodule |  |
| PCDR | 0 | 0 | 0 | 0 | 0 | 0 | 0 | 0 | 1 | 1 | Development, Programmed cell death |  |
| PSRE | 3 | 3 | 0 | 4 | 5 | 1 | 0 | 2 | 1 | 3 | Development, Pollen specific |  |
| RAV5 | 0 | 0 | 0 | 0 | 0 | 1 | 1 | 0 | 1 | 0 | Development, Root, Flower, BR |  |
| REMF | 0 | 1 | 0 | 0 | 1 | 0 | 0 | 1 | 0 | 1 | Development, Flower |  |
| ROOT | 1 | 1 | 1 | 0 | 0 | 0 | 0 | 0 | 0 | 0 | Development, Root hair specific |  |
| SBPD | 4 | 0 | 2 | 0 | 0 | 0 | 1 | 0 | 2 | 0 | Development, Flower |  |
| SCAP | 1 | 1 | 2 | 2 | 2 | 2 | 0 | 1 | 0 | 0 | Development |  |
| SRSF | 0 | 0 | 0 | 0 | 0 | 0 | 0 | 0 | 0 | 4 | Development, Auxin |  |
| TCPF | 0 | 0 | 1 | 0 | 1 | 0 | 0 | 0 | 1 | 0 | Development, ABA, Auxin, GA |  |
| TOEF | 4 | 4 | 1 | 0 | 2 | 3 | 1 | 1 | 2 | 1 | Development |  |
| Sum | 53 | 50 | 56 | 42 | 41 | 51 | 38 | 42 | 38 | 48 |  |  |

Supporting table 2 continued:

| **Matrix Family** | **pCYP79D1** | **pCYP79D2** | **pCYP71E7** | **pCYP7E11** | **pUGT85K4** | **pUGT85K5** | **pLinamarase** | **pHNL10** | **pRubSS** | **pLOX2** | **Description** | **Category** |
| --- | --- | --- | --- | --- | --- | --- | --- | --- | --- | --- | --- | --- |
| CCAF | 14 | 6 | 8 | 10 | 12 | 19 | 16 | 19 | 14 | 21 | Circadian, Auxin, JA, GA | Circadian |
| IBOX | 3 | 5 | 3 | 4 | 5 | 5 | 3 | 1 | 7 | 3 | Light, Circadian |  |
| MYBS | 9 | 5 | 4 | 5 | 5 | 1 | 7 | 6 | 1 | 9 | Circadian |  |
| TDTF | 1 | 0 | 2 | 0 | 0 | 0 | 0 | 1 | 0 | 0 | Circadian |  |
| TODS | 1 | 1 | 1 | 0 | 0 | 2 | 1 | 0 | 0 | 1 | Circadian |  |
| Sum | 28 | 17 | 18 | 19 | 22 | 27 | 27 | 27 | 22 | 34 |  |  |
| GAPB | 4 | 1 | 1 | 5 | 1 | 2 | 4 | 4 | 2 | 1 | Light | Light |
| GTBX | 25 | 22 | 20 | 11 | 30 | 34 | 25 | 25 | 10 | 19 | Light |  |
| HEAT | 3 | 4 | 3 | 6 | 6 | 3 | 0 | 1 | 6 | 8 | Light, heat, ROS |  |
| LREM | 5 | 4 | 1 | 4 | 3 | 3 | 2 | 5 | 2 | 8 | Light |  |
| MYCL | 5 | 5 | 7 | 0 | 1 | 3 | 0 | 0 | 2 | 1 | Light, JA, BR |  |
| PREM | 0 | 0 | 0 | 0 | 0 | 1 | 0 | 0 | 0 | 0 | Light |  |
| Sum | 42 | 36 | 32 | 26 | 41 | 46 | 31 | 35 | 22 | 37 |  |  |
| IDRS | 0 | 1 | 0 | 0 | 0 | 0 | 0 | 0 | 0 | 0 | Iron | Nutrient |
| MYCS | 0 | 0 | 0 | 4 | 1 | 0 | 0 | 0 | 1 | 0 | Phosphate |  |
| OPAQ | 4 | 5 | 2 | 0 | 1 | 2 | 1 | 2 | 3 | 0 | Nutrient, Starvation, Sucrose |  |
| STKM | 2 | 7 | 3 | 1 | 5 | 6 | 5 | 6 | 3 | 7 | Nutrient, Tuber specific, Sucrose |  |
| SUCB | 5 | 8 | 5 | 5 | 8 | 7 | 9 | 4 | 4 | 10 | Nutrient, Sucrose |  |
| SURE | 0 | 0 | 0 | 1 | 0 | 0 | 0 | 0 | 0 | 0 | Nutrient, Sucrose |  |
| TELO | 0 | 0 | 1 | 0 | 0 | 0 | 1 | 0 | 0 | 1 | Nutrient, Cd response |  |
| VRES | 1 | 0 | 1 | 0 | 0 | 0 | 1 | 0 | 0 | 0 | Nutrient, Sulfate |  |
| ZFAT | 1 | 0 | 0 | 0 | 1 | 2 | 1 | 0 | 0 | 0 | Nutrient, Phosphate |  |
| Sum | 13 | 21 | 12 | 11 | 16 | 17 | 18 | 12 | 11 | 18 |  |  |

Figure S1: Linamarin content in 1st unfolded leaf of 3-months-old cassava plants, grown in greenhouse. Samples were collected every hour for 24 h. The experiment was conducted in wintertime in Denmark and artificial light was supplied to ensure light influx during the entire light cycle period. For each time point, two plants with three technical replicates from each were harvested. Bars indicate standard error.

Figure S2: Linamarin content in 1st unfolded leaf of 3-months-old cassava plants, grown in greenhouse. Samples were collected every hour for 48 h. The experiment was conducted in spring in Denmark and artificial light was supplied to ensure light influx during the entire light cycle day period. For each time point three plants with three technical replicates were harvested. Bars indicate standard error.
